# In Vitro Transcribed mRNA Immunogenicity Induces Chemokine‐Mediated Lymphocyte Recruitment and Can Be Gradually Tailored by Uridine Modification

**DOI:** 10.1002/advs.202308447

**Published:** 2024-03-16

**Authors:** Norman M. Drzeniek, Nourhan Kahwaji, Samira Picht, Ioanna Maria Dimitriou, Stephan Schlickeiser, Hanieh Moradian, Sven Geissler, Michael Schmueck‐Henneresse, Manfred Gossen, Hans‐Dieter Volk

**Affiliations:** ^1^ Charité – Universitätsmedizin Berlin, corporate member of Freie Universitaet Berlin and Humboldt‐Universitaet zu Berlin Institute of Medical Immunology Augustenburger Platz 1 13353 Berlin Germany; ^2^ Berlin Institute of Health at Charité – Universitätsmedizin Berlin BIH Center for Regenerative Therapies (BCRT) Föhrer Straße 15 13353 Berlin Germany; ^3^ Berlin‐Brandenburg School for Regenerative Therapies (BSRT; graduate school 203 of the German Excellence Initiative) Augustenburger Platz 1 13353 Berlin Germany; ^4^ Julius Wolff Institute (JWI) Berlin Institute of Health at Charité – Universitätsmedizin Berlin Augustenburger Platz 1 13353 Berlin Germany; ^5^ Department of Biology, Chemistry, Pharmacy, Institute of Chemistry and Biochemistry Freie Universität Berlin Thielallee 63 14195 Berlin Germany; ^6^ CheckImmune GmbH Campus Virchow Klinikum Augustenburger Platz 1 13353 Berlin Germany; ^7^ Institute of Active Polymers Helmholtz‐Zentrum Hereon Kantstraße 55 14513 Teltow Germany; ^8^ Berlin‐Brandenburg Center for Regenerative Therapies (BCRT) Augustenburger Platz 1 13353 Berlin Germany; ^9^ Charité – Universitätsmedizin Berlin, corporate member of Freie Universität Berlin and Humboldt‐Universität zu Berlin Berlin Center for Advanced Therapies (BeCAT) Augustenburger Platz 1 13353 Berlin Germany

**Keywords:** 5‐methoxyuridine, CXCL10, immunoengineering, innate immune activation, N1‐methylpseudouridine, nucleoside‐modified messenger RNA, T cell migration

## Abstract

Beyond SARS‐CoV2 vaccines, mRNA drugs are being explored to overcome today's greatest healthcare burdens, including cancer and cardiovascular disease. Synthetic mRNA triggers immune responses in transfected cells, which can be reduced by chemically modified nucleotides. However, the side effects of mRNA‐triggered immune activation on cell function and how different nucleotides, such as the N1‐methylpseudouridine (m1Ψ) used in SARS‐CoV2 vaccines, can modulate cellular responses is not fully understood. Here, cellular responses toward a library of uridine‐modified mRNAs are investigated in primary human cells. Targeted proteomics analyses reveal that unmodified mRNA induces a pro‐inflammatory paracrine pattern marked by the secretion of chemokines, which recruit T and B lymphocytes toward transfected cells. Importantly, the magnitude of mRNA‐induced changes in cell function varies quantitatively between unmodified, Ψ‐, m1Ψ‐, and 5moU‐modified mRNA and can be gradually tailored, with implications for deliberately exploiting this effect in mRNA drug design. Indeed, both the immunosuppressive effect of stromal cells on T‐cell proliferation, and the anti‐inflammatory effect of IL‐10 mRNA are enhanced by appropriate uridine modification. The results provide new insights into the effects of mRNA drugs on cell function and cell‐cell communication and open new possibilities to tailor mRNA‐triggered immune activation to the desired pro‐ or anti‐inflammatory application.

## Graphical abstract

Synthetic mRNA can serve as a template for protein overexpression but also as an immune stimulant. Unmodified mRNA triggers anti‐viral‐like responses in transfected cells. It induces the secretion of lymphocyte‐attracting chemokines, which recruit T and B lymphocytes. This effect can be gradually tailored by modifying the uridine chemistry, which creates opportunities for deliberately exploiting immune activation in mRNA drugs.

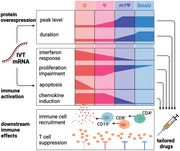

## 1 Introduction

In vitro transcribed (IVT) mRNA has entered the pharmaceutical landscape as a new class of drugs with unprecedented momentum, having rapidly demonstrated its utility as a successful vaccination technology in a broad human population during the SARS‐CoV2 pandemic.^[^ ^1^ ^]^ In addition to vaccinating against infectious diseases, the therapeutic potential of mRNA is being intensively explored to address some of today's greatest healthcare challenges, including cancer^[^ ^2^, ^3^ ^]^ and cardiovascular regeneration.^[^ ^4^, ^5^ ^]^

IVT mRNA is a highly versatile tool for the manipulation of cells and tissues. It can act as a template for the production of virtually any cell‐secreted protein, such as cytokines^[^ ^6^ ^]^ and growth factors,^[^ ^7^ ^]^ transcription factors that control gene expression,^[^ ^8^ ^]^ as well as transmembrane proteins, including receptors for migration^[^ ^6^ ^]^ or recognition of (cancer) antigens.^[^ ^9^, ^10^ ^]^ Compared to DNA‐based genetic engineering, the use of IVT mRNA to manipulate the function of cells and tissues has several advantages. Unlike DNA, mRNA does not need to enter the cell nucleus in order to be translated into the protein of interest. This leads to rapid and efficient transfection of cells, but is also the most important safety advantage of IVT mRNA over traditional gene therapy: because it does not integrate into the genome, the use of mRNA eliminates the risk of disrupting vital genes or causing cancer‐promoting mutations.^[^ ^11^, ^12^ ^]^

Despite its potential for clinical applications, IVT mRNA technology also faces some challenges that have dampened the enthusiasm toward its therapeutic use. One challenge is the rather short‐lived effective expression window, usually lasting only one or two days, due to the low stability and rapid degradation of the mRNA.^[^ ^13^, ^14^ ^]^ Another major limitation of IVT mRNA is the recognition of IVT mRNA as foreign by intracellular pattern recognition receptors such as TLR 7 and 8, RIG‐1 or MDA‐5, which triggers an inflammatory type I interferon response in transfected cells, similar to viral RNA (Figure 2 schematic). This triggers several cellular defense mechanisms, such as faster degradation of mRNA, translational arrest and even apoptosis to inhibit viral replication and prevent the spread of infection.^[^ ^15^ ^]^ The extent to which these endogenous defense mechanisms also affect the functionality of IVT mRNA‐engineered cells is still incompletely understood, e.g., in the case of ex vivo modified cell therapeutics^[^ ^6^, ^16^, ^17^ ^]^ or in transfected tissues upon in situ delivery of mRNA drugs.^[^ ^4^, ^18^ ^]^

Karikó and Weissman discovered that the intracellular immune response toward IVT mRNA can be reduced by replacing uridine contained in the IVT mRNA by chemically modified derivates, such as pseudouridine (Ψ).^[^ ^19^, ^20^ ^]^ This discovery paved a way toward unleashing the therapeutic potential of mRNA drugs, recently gaining its pioneers the Nobel Prize in Medicine. Since the first studies on Ψ‐modified mRNA, the landscape of uridine modifications has evolved. Most notably, N1‐methylpseudouridine (m1Ψ), which was used in the SARS‐CoV2 vaccines tozinameran (BioNTech‐Pfizer) and elasomeran (Moderna),^[^ ^1^, ^21^ ^]^ was shown to reduce immune activation, improve mRNA translation and yield higher protein production than Ψ.^[^ ^22^, ^23^ ^]^ Another uridine analogue, 5‐methoxyuridine (5moU), was shown to prolong the expression of IVT mRNA, thus addressing the other major limitation of mRNA drugs.^[^ ^13^ ^]^ However, the studies which proposed those new nucleotides characterized them using immortalized cell lines, which possess a survival advantage over primary human tissue cells, exhibit an altered intracellular signaling, and lack cell type‐specific functions. This makes it difficult to assess cell fitness and immune‐related changes in their phenotype and function. Furthermore, a side‐by‐side comparison of the different uridine modifications is lacking.

Here we aim to systematically characterize the expression and immune activation of IVT mRNA equipped with Ψ, m1Ψ, 5moU and unmodified uridine (U) in patient‐derived human cells and to understand potential immune‐related side effects. We hypothesize that the cell‐cell communication of mRNA transfected cells might be altered, thus amplifying the cascade of mRNA‐triggered inflammation beyond the originally targeted cells.

As a model to study cellular responses, we use bone marrow stromal cells (BMSCs) derived from explanted hip bones of patients who underwent hip replacement. Not only is this cell type generally well‐studied,^[^ ^24^ ^]^ allowing for ample comparison and generalization of our data, but has also been a target for IVT mRNA‐based cell engineering in a variety of preclinical studies, which used transfected BMSCs to deliver mRNA‐overexpressed cytokines for therapeutic purposes.^[^ ^6^, ^14^, ^25^ ^]^ In most such studies, the intended application of the mRNA‐modified BMSCs is to resolve inflammation, often proposing interleukin‐10 (IL‐10), a potent anti‐inflammatory cytokine^[^ ^26^ ^]^ as the mRNA‐encoded target. At first glance, it would appear paradoxical to use IVT mRNA for anti‐inflammatory indications, considering that IVT mRNA itself can give rise to pro‐inflammatory reactions. Importantly, none of those anti‐inflammatory application studies characterized the cellular immune response toward the introduced mRNA or any potential impact of mRNA nucleotide chemistry on the fitness and immunomodulatory function of the transfected cells.

**Scheme 1:**
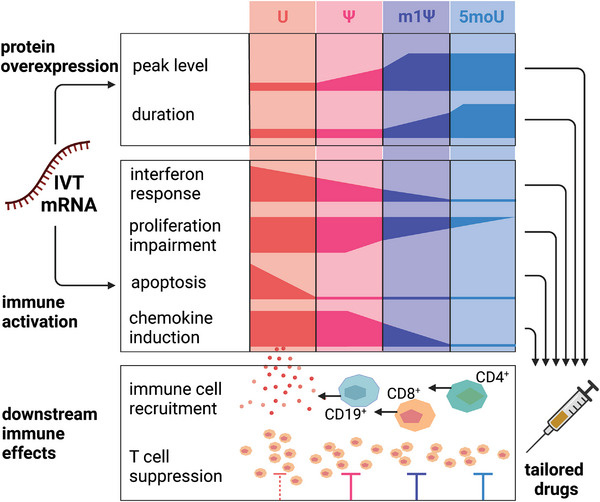
Study concept and overview. Synthetic mRNA has not one, but two effects on cells: it serves as a template for protein overexpression but also as an immune stimulatory agent. We find that in addition to affecting a range of other cell functions, unmodified mRNA triggers an anti‐viral‐like response in transfected cells and induces a pro‐inflammatory paracrine pattern marked by the secretion of lymphocyte‐attracting chemokines. This mRNA‐induced secretome pattern recruits both T and B lymphocytes, which bears implications and opportunities for consciously exploiting this effect in mRNA drugs.

We characterize in depth how the immune activation by IVT mRNA impacts the fitness and function of transfected cells and to what extent different uridine modifications are effective at reducing the mRNA's footprint on cell phenotype and function (**Scheme** **1**). We place a special emphasis on whether mRNA immunogenicity triggers inflammatory paracrine signaling toward other cells, as this would spread the consequences of mRNA‐related side effects beyond the transfected cell or tissue. Using IL‐10 mRNA as a therapeutically relevant example, we investigate whether immune activation triggered by mRNA diminishes the mRNA's suitability for immune modulatory applications. Importantly, we ask to what extent the use of recent mRNA modifications, such as the m1Ψ used in SARS‐CoV2 vaccines, could restore this suitability.

Importantly, we show that the extent of these mRNA‐triggered changes in cell function varies quantitatively between unmodified, Ψ‐, m1Ψ‐, and 5moU‐modified IVT mRNA (from highest immune activation to lowest). Thus, the intensity of mRNA's immune stimulatory function can be gradually tailored to the desired inflammatory (e.g., cancer or viral vaccination) or non/anti‐inflammatory (e.g., tolerance induction, protein replacement, regeneration) application.

To illustrate this, we show that both the anti‐proliferative effect of BMSCs on T cell proliferation, and the immunosuppressive effect of IL‐10 mRNA could be reinforced through uridine modification.

## 2 Results

### 2.1 In Vitro Transcribed mRNA Yields High and Lasting Expression, Depending on Uridine Chemistry

**Figure 1:**
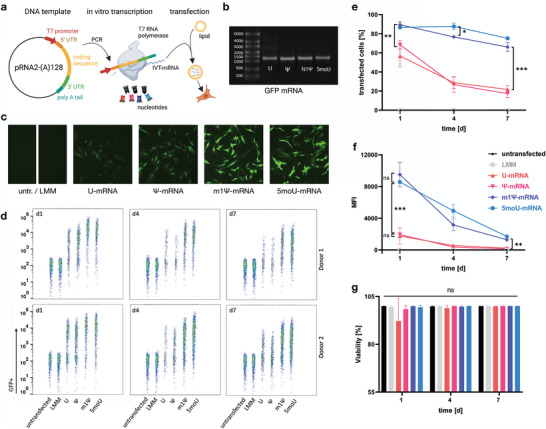
Chemical uridine modifications differently affect mRNA expression levels and kinetics. a) A linearized plasmid containing the coding sequence of interest was used as a template for mRNA in vitro transcription, incorporating the different chemically modified nucleotide triphosphates. This IVT‐mRNA was complexed with cationic lipid and introduced into BMSCs. b) The IVT‐mRNA was run on an agarose gel to exclude impurities and ensure integrity prior to transfection. Single bands are visible for all four transcripts of GFP‐mRNA containing different uridine analogues. c) At day 1 post transfection, BMSCs displayed different levels of GFP fluorescence depending on the mRNA used. d–f) The transfection efficacy and expression levels (MFI = mean fluorescence intensity) was quantified using flow cytometry 1, 3 and 7 days after transfection. m1Ψ‐ and 5moU‐modified mRNA resulted in higher transfection efficacy, expression peaks and durations compared to U‐ or Ψ‐mRNA. Using the same data as input, d) is a dot plot showing the distribution of GFP signal at single cell resolution for one sample of each tested biological donor, e) visualizes the mean percentage of GFP^+^ cells and f) summarizes the changes in MFI over time. g) mRNA transfection did not reduce cell viability. Only U‐mRNA resulted in a tendential viability drop 1 day after transfection. Data in e)‐g) presented as mean ± SD, n = 4. P‐values are calculated using two‐way ANOVA with Tukey's post‐hoc test, * p<0,05; ** p<0,01; *** p<0001; ns = not significant.

A linear fragment was amplified from the pRNA2‐(A)128 plasmid containing the coding sequence for EGFP. This fragment extended from the T7 promoter to the 128‐base polyadenine tail. The PCR product was used as a template to transcribe the mRNA in vitro (**Figure** **1**a). Four mRNAs were synthesized containing different uridine analogues: pseudouridine (Ψ), N1‐methylpseudouridine (m1Ψ), 5‐methoxyuridine (5moU) and unmodified uridine itself (U). All synthesized mRNAs showed excellent integrity and were of the expected size (Figure 1b). The mRNAs were complexed with lipofectamine (LMM) and delivered to BMSC culture (Figure 1a).

As observed by fluorescence microscopy, EGFP was expressed in all mRNA‐transfected groups at 24 h post‐transfection (Figure 1c). However, there were strong differences in expression levels and numbers of transfected cells between groups. m1Ψ and 5moU mRNA resulted in stronger EGFP expression than U and Ψ mRNA. The cells were detached, and the expression was quantified by flow cytometry, which confirmed the trend observed (Figure 1d). Quantification of EGFP+ cells (Figure 1e) showed that all mRNAs were successfully expressed over the course of one week (days 1, 4 and 7 post‐transfection). However, m1Ψ and 5moU mRNA resulted in >80% of transfected cells, whereas the proportion of EGFP+ cells in the U and Ψ groups did not exceed 80% and declined rapidly to less than 30% by day 7. Remarkably, even after 1 week in culture, >60% of m1Ψ‐ and 5moU‐transfected cells were still EGFP^+^. EGFP fluorescence intensity was also measured over time (Figure 1f), representing the intensity of mRNA expression per transfected cell. Here, m1Ψ‐mRNA and 5moU‐mRNA gave the strongest fluorescence peak at 24 h post‐transfection, but the signal intensity in m1Ψ‐mRNA ‐transfected cells decreased rapidly (<50% intensity on day 4). In contrast, for 5moU‐mRNA fluorescence declined more slowly (>50% intensity on day 4). At day 7 post‐transfection, EGFP signal was still observed at ≈20% of the day 1 signal intensity for both m1Ψ and 5moU mRNA. The signal intensity in the U and Ψ groups was >4‐fold lower than 5moU or m1Ψ on day 1. It decreased rapidly and was also significantly lower on day 7. The viability of transfected BMSCs was not significantly reduced by any of the mRNAs. However, there was a tendential, although not significant, initial drop in viability upon transfection with U‐mRNA (Figure 1g).

### 2.2 Lasting Impairment of Cell Fitness by Unmodified or Ψ‐Modified mRNA

Cells can recognize exogenous RNA introduced during viral infections.^[^ ^27^ ^]^ Detection of immunogenic RNA by innate immune receptors such as TLR 7 and 8, RIG‐1 or MDA‐5 is known to trigger a type I interferon response that can potentially lead to translational arrest,^[^ ^15^, ^20^, ^28^ ^]^ accelerated RNA degradation^[^ ^29^ ^]^ and even apoptosis of the infected (or transfected) cell.^[^ ^30^, ^31^ ^]^ Thus, several aspects potentially associated with an antiviral immune response were subsequently investigated to elucidate the impact of IVT mRNA on the fitness and function of transfected cells.

**Figure 2:**
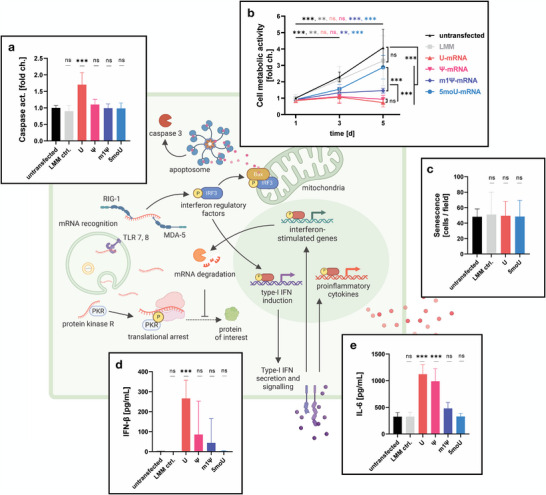
IVT‐mRNA immunogenicity affects phenotype and fitness of transfected cells. White panels display primary data collected for BMSCs. The green background schematic illustrates corresponding known anti‐viral responses against endogenous mRNA, which are further explained in the introduction. a) Compared to untransfected BMSCs and the lipofectamin‐only control (LMM ctrl.), unmodified EGFP mRNA (U) induced a significant increase in caspase activity. n = 8, P‐values are calculated using one‐way ANOVA with Dunnett's post‐hoc test for many‐to‐one comparisons. b) Cell metabolic activity was traced over 5 days post‐transfection to quantify cell proliferation. U‐ and Ψ‐mRNA impaired cell proliferation. n = 8, except d5: n = 12, mixed‐effects analysis with Tukey's post‐hoc test for multiple comparisons. c) mRNA did not induce senescence in transfected cells. Cultures were stained for senescence‐associated β‐galactosidase on day 5 post‐transfection and senescent cells were quantified. n = 6, one‐way ANOVA with Dunnett's post‐hoc test. d) IFN‐β secretion was induced by mRNA transfection, especially with U‐mRNA. In untransfected cells or the 5moU group no IFN‐β was detected in cell supernatants. n = 9, one‐way ANOVA with Dunnett's post‐hoc test. e) IL‐6 secretion was increased by mRNA transfection, especially with U‐mRNA and Ψ‐mRNA. Significance in a, d, e is shown in relation to untransfected BMSCs. n = 9, one‐way ANOVA with Dunnett's post‐hoc test. All data presented as mean ± SD. * p<0,05; ** p<0,01; *** p<0001; ns = not significant.

Unmodified U‐mRNA significantly increased caspase activity, compared to untransfected BMSCs, while the chemically modified mRNAs (or the LMM without any mRNA) did not (**Figure** **2**a). To understand if mRNA transfection causes a lasting impairment of cell proliferation, metabolic activity of BMSCs was measured on days 1, 3 and 5 post‐transfection (Figure 2b). Although no differences in metabolic activity could be discerned 24 h after transfection, cells transfected with U or Ψ‐mRNA failed to proliferate, indicating a lasting impairment of cell fitness. At day 5, metabolic activity in the m1Ψ and 5moU groups was significantly higher than in the U and Ψ groups. Remarkably, 5moU‐transfected BMSCs proliferated to a similar extent as untransfected cells or the LMM control group, implying a minimal impact of this IVT‐mRNA on cell fitness. On day 5 post‐transfection, cells were stained for senescence‐associated β‐galactosidase activity. Similar levels of senescence could be observed in all tested groups, indicating that this parameter is not affected by mRNA transfection, regardless of its immunogenicity (Figure 2c).

### 2.3 Uridine Derivates Result in Distinct Immunogenicity of IVT‐mRNA

To confirm the innate immune activation‐related mechanism of the observed differences, the secretion of type I interferon (IFN‐β), which is at the nexus of mRNA‐triggered immune activation signaling,^[^ ^32^ ^]^ was measured (Figure 2d). Indeed, IFN‐β was detected in the unmodified mRNA (U) and, to a lesser extent, in the Ψ and m1Ψ groups. This suggests that these mRNAs, but not the 5moU mRNA, induce an innate immune response in transfected cells. The increased secretion of the pro‐inflammatory marker IL‐6 by U‐ or Ψ‐mRNA‐transfected BMSCs (Figure 2e) also reflected the immunogenicity of the different mRNAs.

### 2.4 Unmodified mRNA Induces Pro‐Inflammatory Paracrine Shift in Transfected Cells

**Figure 3:**
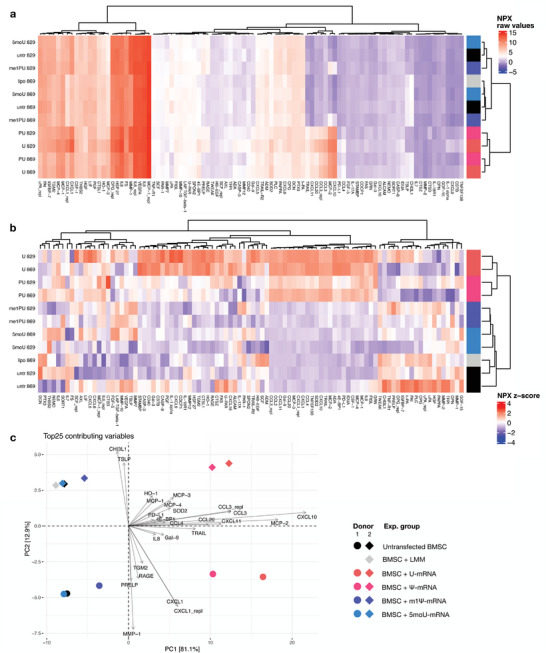
Chemical modification of IVT‐mRNA changes the secretion profile of transfected BMSCs. a) Secretome profiling of mRNA‐transfected and untransfected BMSCs. A cluster of cytokines was strongly induced by U‐ and Ψ‐mRNA. Raw NPX values are shown. b) shows the same data but differences between analyte concentrations in experimental groups are shown as fold change for each individual analyte. Complex shifts in the paracrine pattern were induced by U‐mRNA and by Ψ‐mRNA, irrespective of difference in MSC donor. The secretome pattern of 5moU‐ or m1Ψ‐transfected BMSC and LMM (Lipofectamine Messenger Max) treated BMSCs clustered with that of untreated cells. c) The biplot shows loading vectors (arrows) of the top 25 analytes which contributed most strongly to the total variation within the data. It is overlaid with scores (colored dots and diamonds representing experimental groups) for the two highest ranking principal components of data variation within the principal component analysis (PCA). The PCA reveals that primarily the secretion of chemokines in the U‐ and Ψ‐mRNA groups ‐and to a lesser degree in the m1Ψ group‐ contributed to differences between the different secretomes, with CXCL10 being the most strongly induced factor. Biological differences between BMSC donors (PC2) accounted for 12.9% of the overall data variation. The effect of mRNAs (PC1) was orthogonal to donor differences and comparable in both cell donors. n = 2 cell donors.

It is well known that BMSCs secrete a large number of growth factors and cytokines that regulate the BM niche and that constitute the mode of action of therapeutically transplanted BMSCs. We hypothesized that transfection with mRNAs containing different uridine modifications might also affect the profile of cell‐secreted paracrine mediators, based on the differential immunogenicity of different IVT mRNAs and their observed effects on cellular phenotype. Culture supernatants collected 24 h post‐transfection from BMSCs of two different patient donors were subjected to an array of >250 proteins (**Figure** **3**a). 94 analytes were detected across all groups. All secretomes contained relatively high levels of vascular endothelial growth factor A (VEGFA), a key mediator of angiogenesis^[^ ^33^ ^]^ interleukins 6 and 8 (IL6; IL8), matrix metalloprotease 1 (MMP‐1), monocyte chemoattractant protein‐1 (MCP‐1) and follistatin (FS), a muscle growth factor.^[^ ^34^ ^]^

Strikingly, a cluster of chemokines was noticeably upregulated in the secretome of U‐mRNA and Ψ‐mRNA transfected BMSCs. These included C‐X‐C motif chemokine ligands 10 and 11 (CXCL10; CXCL11), which both attract activated T lymphocytes through the chemokine receptor CXCR3,^[^ ^35^ ^]^ C‐C motif chemokine ligands 3 and 20 (CCL3; CCL20), monocyte chemoattractant protein 2 (MCP‐2), and TNF‐related apoptosis‐inducing ligand (TRAIL).

A comparison of the relative regulation of each analyte between the different groups allowed a more detailed assessment of secretome regulation (Figure 3b). This analysis revealed a larger cluster of chemokines that were upregulated by the U‐mRNA and, to a slightly lesser extent, by the Ψ‐mRNA in both cell donors. This cluster included the upregulation of IL8 in addition to the secretome shift seen in Figure 3a. In addition, there was a cluster of analytes that were primarily upregulated by U‐mRNA, but not by Ψ‐mRNA. These included C‐X‐C motif chemokine ligand 9 (CXCL9), another ligand of CXCR3, and caspases 3 and 8, in line with Figure 2a.

### 2.5 Inflammatory Secretome Shifts Induced by Different Uridine Analogues Follow a Gradual Trend

To measure the relationship between the secretomes induced in response different mRNA chemistries, also considering conceivable biological differences between patient‐derived human cells, the principal components of the overall data variation were determined (Figure 3c). The principal component analysis (PCA) revealed that differences between experimental groups (i.e., mRNA chemistries) accounted for 81.1% of the overall data variation (PC1). Biological differences between BMSC donors (PC2) accounted for 12.9% of the overall data variation. The effect of mRNA treatment was orthogonal to donor differences and comparable (parallel) in both cell donors.

Primarily the secretion of chemokines contributed to differences between the different secretomes, with CXCL10 being the most strongly induced factor. The relationship between the secretomes induced by different mRNA chemistries followed a gradual trend along the dominant variable vector “CXCL10”: U, Ψ and m1Ψ (from the strongest to the weakest shift), with group scores forming two parallel lines along the most dominant vectors (one for each donor). Remarkably, the secretome of 5moU‐mRNA transfected cells clustered with untransfected cells, indicating no detectable secretome shift induced by this mRNA.

### 2.6 Cells Transfected with Unmodified mRNA Attract T and B Lymphocytes

**Figure 4:**
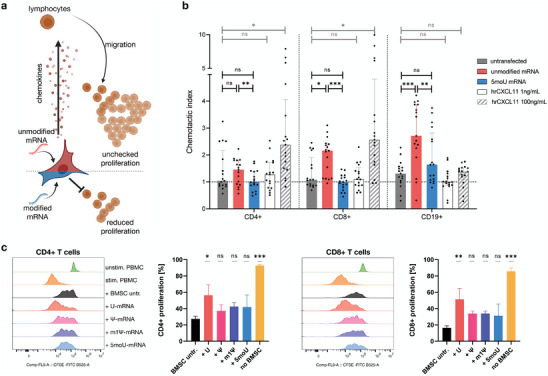
BMSCs transfected with unmodified mRNA attract lymphocytes and display reduced immunomodulatory function. a) Schematic summary of the investigated crosstalk between transfected BMSCs and immune cells. b) PBMC migration was stimulated with BMSC‐conditioned media. BMSCs transfected with unmodified mRNA attracted CD4^+^ and CD8^+^ T cells and CD19^+^ B cells. These effects were not observed for untransfected BMSCs or BMSCs transfected with 5moU‐mRNA. Data presented as median with interquartile range, n = 17. P‐values are calculated using RM one‐way ANOVA with Tukey's post‐hoc test. c) CFSE‐stained PBMCs were polyclonally stimulated and co‐cultured with mRNA‐transfected BMSCs for 5 days. Dilution of the CFSE dye by cell division allowed to quantify T cell proliferation using flow cytometry. BMSCs reduced the proliferation of CD4^+^ (left) and CD8^+^ (right) T cells in response to stimulus (histograms). This effect was significantly impaired for U‐mRNA‐transfected BMSCs (bar graphs). Significance is shown in comparison to untransfected BMSCs. Data presented as mean ± SD, n = 3, one‐way ANOVA with Tukey's post‐hoc test. * p<0,05; ** p<0,01; *** p<0001; ns = not significant.

We hypothesized that the secretome shift induced by unmodified mRNA would lead to immune cell recruitment toward mRNA‐transfected cells, since CXCL9, 10 and 11, a functional cluster of highly induced chemokines, are ligands for the migration receptor CXCR3 found on T lymphocytes (**Figure** **4**a).

Primary immune cells (peripheral blood mononuclear cells; PBMCs) from freshly collected human donor blood were allowed to migrate across a porous membrane toward a gradient of BMSC secretome (Figure 4b). Human recombinant CXCL11 was used as a positive control for the migration of T cells and fresh culture medium was used as a blank. Compared to untransfected or 5moU‐mRNA transfected BMSCs, the secretome of U‐mRNA transfected BMSCs induced a pronounced T lymphocyte recruitment. In particular, the migration of CD8^+^ T cells was significantly upregulated by U‐mRNA transfected BMSCs in comparison to all other groups. Surprisingly, CD19^+^ B lymphocytes, which are not a primary target of CXCR family chemokines, showed very strong migration. B cells migrated strongly toward the U‐mRNA secretome and slightly toward the untransfected and 5moU‐mRNA secretomes. Even high doses of recombinant CXCL11 only led to a slight but non‐significant increase in B cell attraction.

### 2.7 Transfection with Unmodified mRNA Impairs the Immunomodulatory Function of BMSCs

BMSCs are applied clinically for their immune modulatory potential and are known to suppress proliferation of activated T cells in vitro.^[^ ^24^, ^36^ ^]^ Next, we wondered whether the changes in the interaction between mRNA‐transfected BMSCs and lymphocytes would be limited to migration and whether the mRNA‐transfected cells would be able to suppress or rather promote the proliferation of the lymphocytes they recruit (Figure 4a).

Proliferation of CFSE‐labelled primary human PBMCs co‐cultured with untransfected versus mRNA‐transfected BMSCs for 5 days was quantified by measuring the dilution of the CFSE signal with each cell division (Figure 4c, histograms). After polyclonal activation, more than 80% of the T cells proliferated strongly in monoculture. In co‐culture, untransfected BMSCs strongly reduced the proliferation of T cells to <30% and <20% for CD4^+^ and CD8^+^ cells, respectively. However, this effect was significantly impaired when BMSCs were transfected with U‐mRNA. Approximately half of the CD4^+^ or CD8^+^ T cells proliferated strongly (Figure 4c, bar graphs). The immunomodulatory function of BMSCs was not significantly affected by transfection with nucleotide‐modified mRNAs (U, Ψ and m1Ψ).

### 2.8 Overexpression of mRNA‐Encoded IL‐10 in BMSCs

A common use for IVT mRNA technology in BMSCs is the overexpression of anti‐inflammatory agents, such as IL‐10 on top of the cells' natural immune modulatory functions.^[^ ^6^, ^25^ ^]^ As none of these studies investigated the effect of the immunogenicity of the mRNA on the phenotype of the BMSCs or their immunomodulatory potential, we hypothesized that the use of immunogenic unmodified mRNA might counteract the intended anti‐inflammatory effect of the overexpressed IL‐10.

**Figure 5:**
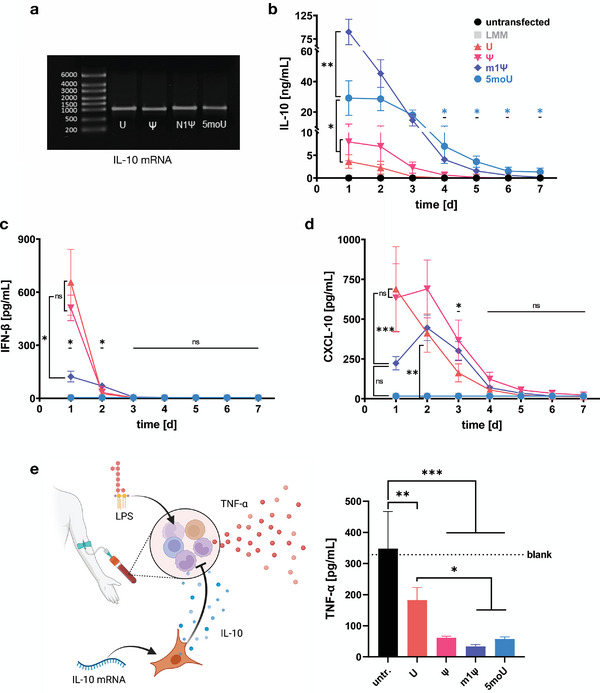
mRNA nucleotide chemistry determines the effectiveness of IL‐10 overexpressing BMSCs in sepsis attenuation model. a) IVT‐mRNA coding for IL‐10 shows high integrity and purity. b) IL‐10 was secreted from transfected BMSCs for up to 6 days (significance shown for 5moU). U‐ and Ψ‐mRNA yielded lower and shorter expression than m1Ψ‐ and 5moU‐mRNA. n ≥ 6. P‐values are calculated using mixed‐effects analysis with Tukey's post‐hoc test. c)‐d) IFN‐β and CXCL10 secretion were measured to assess the immune response against IL‐10‐mRNA. INF‐β was induced by U‐, Ψ‐, and m1Ψ‐mRNA and peaked 1 day post transfection. CXCL10 was secreted in the U‐, and Ψ‐mRNA groups ‐and to a lesser degree in the m1Ψ‐mRNA group‐ until up to 3 days post‐transfection. n = 4, two‐way ANOVA with Tukey's post‐hoc test. e) The efficacy of BMSC‐overexpressed IL‐10 was assessed in a sepsis attenuation model. Human donor blood was challenged with lipopolysaccharide (LPS) and TNF‐α secretion was measured in response. IL‐10 from transfected BMSCs was hypothesized to attenuate the TNF‐α response (schematic). Compared to untransfected BMSCs, all IL‐10 secreting BMSC groups reduced the TNF‐α response. This was significantly less effective in BMSCs transfected with U‐mRNA compared to the m1Ψ and 5moU mRNAs. n = 4, two‐way ANOVA with Tukey's post‐hoc test. All data presented as mean ± SD. * p<0,05; ** p<0,01; *** p<0001; ns = not significant.

IL‐10 mRNA was transcribed in vitro (**Figure** **5**a) and introduced into BMSCs. All of the mRNAs led to a level of IL‐10 secretion in the nanogram per milliliter range (Figure 5b). The levels and duration of IL‐10 production for the respective uridine chemistries were consistent with the trend observed for EGFP mRNA, with m1Ψ‐mRNA resulting in the highest peak expression and 5moU‐mRNA resulting in the longest expression of all mRNAs for up to 6 days. The type I interferon response and chemokine induction observed for the different EGFP mRNA groups were confirmed by monitoring IFN‐β and CXCL10 secretion: IFN‐β secretion was induced by all mRNAs except 5moU‐mRNA, with U and Ψ mRNAs stimulating IFN‐β production more than m1Ψ‐mRNA (Figure 5c). A similar relationship between mRNA groups was observed for CXCL10 secretion on day 1 post‐transfection. Interestingly, CXCL10 secretion in U‐mRNA transfected cells decreased after the initial peak but increased and reached a peak on day 2 for both Ψ‐ and m1Ψ‐mRNA (Figure 5d).

### 2.9 mRNA Immunogenicity Counters the Anti‐Inflammatory Effects of mRNA‐Expressed IL‐10

A sepsis attenuation assay in human blood was performed to determine whether the immunogenicity of the mRNA would counteract the intended anti‐inflammatory effects of IL‐10 overexpression (Figure 5e): Freshly collected blood from healthy donors was stimulated with bacterial endotoxin/lipopolysaccharide (LPS) to induce TNF‐α secretion, as is the case in sepsis.^[^ ^37^, ^38^ ^]^ IL‐10 is known to reduce TNF‐α production by monocytes.^[^ ^39^ ^]^ Therefore, supernatants from IL‐10 mRNA‐transfected BMSCs were added to LPS‐stimulated blood. TNF‐α was measured. All BMSC groups overexpressing IL‐10 reduced TNF‐α production in human blood. This confirmed the bioactivity of the mRNA‐encoded IL‐10. However, the BMSCs transfected with 5moU and m1Ψ mRNA were significantly more effective in attenuating the septic response than the unmodified mRNA group.

### 2.10 The Relationship between Nucleotide Chemistry and Immune Activation is Independent of the mRNA Coding Sequence

**Figure 6:**
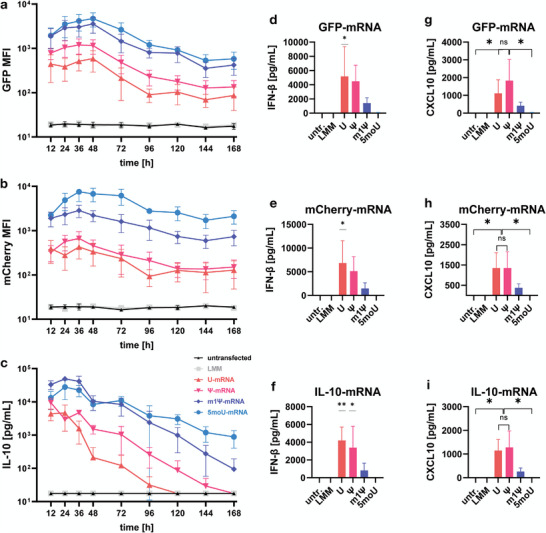
Generalizability of cellular responses to mRNA with different coding sequences. BMSCs were transfected side by side using 3 sets of IVT‐mRNAs coding for EGFP, mCherry or IL‐10. Expression kinetics of the target protein a–c), immune activation represented by the induction of IFN‐β d–f), and chemokine induction g–i) were compared: a,b) The mean fluorescence intensity (MFI) kinetics of the two different fluorescence proteins were quantified using flow cytometry and are closely comparable. c) Secreted IL‐10 was measured in supernatants and shows a similar relationship between mRNA modifications. d–f) The relationship between nucleotide chemistry and IFN‐β induction was closely comparable, regardless of which protein was encoded by the IVT‐mRNA. One‐way ANOVA with Dunnett's post‐hoc test for many‐to‐one comparisons. g–i) The relationship between nucleotide chemistry and CXCL10 induction was closely comparable between mRNAs with different coding sequences. One‐way ANOVA with Tukey's post‐hoc test. All data presented as mean ± SD, all n = 3. * p<0,05; ** p<0,01; *** p<0001; ns = not significant.

To verify to what extent findings obtained from an mRNA coding for one specific protein could be applied to any other mRNA, BMSCs were transfected side‐by‐side with three sets of IVT‐mRNA, coding for EGFP, mCherry or IL‐10 and protein expression, immune activation and chemokine induction were compared. For all three sets of mRNAs, U‐mRNA resulted in the lowest protein expression, while 5moU resulted in the most stable protein expression, especially at later timepoints >72 h post‐transfection (**Figure** **6**a–c). The intracellular fluorescence kinetics of EGFP and mCherry were closely comparable (Figure 6a,b). Secreted IL‐10 was measured in culture supernatants which were changed at each interval and thus shows an earlier expression peak (24 h) and steeper decline due to lack of accumulation, but an overall comparable relationship between the mRNA chemistries (Figure 6c). Similar levels of IFN‐β were detected in supernatants of cells transfected with unmodified EGFP (51.6 ng mL^−1^; Figure 6d), mCherry (68.3 ng mL^−1^; Figure 6e), or IL‐10‐encoding (42.0 ng mL^−1^; Figure 6f) IVT‐mRNA, suggesting that the immunogenicity of mRNA is not determined by the nucleotide sequence of the coding region. Similarly, the U‐mRNAs and Ψ‐mRNAs of all three different mRNA sets induced comparable levels of CXCL10 secretion (Figure 6g–i). The chemokine induction by Ψ‐mRNA was not significantly different from U‐mRNA but significantly higher than in 5moU‐transfected cells.

## 3 Discussion

Unmodified in vitro transcribed mRNA is known to illicit inflammatory innate immune responses in transfected cell. mRNA drugs are being widely explored for a variety of therapeutic scenarios, including vaccines (infectious diseases^[^ ^1^ ^]^ and cancer^[^ ^3^ ^]^), protein replacement and regenerative medicine.^[^ ^4^ ^]^ While in the vaccine context, a certain level of mRNA‐triggered immune activation may be beneficial to the therapeutic effect,^[^ ^3^, ^40^ ^]^ it is certainly undesirable for protein replacement. Also in regenerative processes, a fine balance between early inflammation and timely termination thereof is crucial to the healing outcome.^[^ ^41^, ^42^ ^]^ As such, an understanding of the functional consequences of mRNA immune activation, as well as means to uncouple the immune activating function from the protein template function of mRNA are necessary to tailor mRNA drugs to their respective application. Karikó et al. have shown in their pioneering work that especially the replacement of uridine with pseudouridine (Ψ) reduces immune recognition of IVT mRNA. In primary stromal cells derived from two patient donors, we systematically compared the expression and immune stimulatory effects of mRNA modified with different uridine analogues, including uridine (U), Ψ, as well as the more recently introduced m1Ψ and 5moU.

Initially we showed that the mRNA modifications affect expression levels and kinetics, with m1Ψ yielding the highest peak expression and 5moU resulting in a prolonged expression kinetic. This result is consistent with earlier observations made for those two nucleotide‐modified mRNAs in cell lines.^[^ ^13^, ^22^ ^]^ We observed a continuous drop in the percentage of EGFP^+^ cells 4 and 7 days after transfection with U or Ψ, while the percentage of 5moU‐transfected EGFP^+^ cells remained stable (Figure 1e). This implies a higher stability of 5moU‐mRNA, which is consistent with prior reports.^[^ ^13^ ^]^ When interpreting the data in the context of the initial expression intensity (Figure 1f) a confounding factor could be that as the transfected cells divide, the EGFP protein is diluted out faster in those cells which did not express it strongly to begin with. However, considering the reduced proliferation rate of those cells (Figure 2b), this dilution effect is likely rather moderate.

Based on theoretical knowledge of the intracellular signaling pathways triggered by unmodified mRNA, we characterized phenotypic changes in mRNA‐transfected cells from different angles. We observed that unmodified mRNA, but none of the modified mRNAs increased caspase activity in transfected cells, which is a hallmark of apoptosis (Figure 2a). To understand whether the mRNAs would have a lasting impact on cell fitness, which extends beyond potential transfection‐related stress, we allowed sparsely seeded transfected and untransfected cells to proliferate over a course of 5 days and measured their metabolic activity (Figure 2b). All mRNAs impaired the increase in metabolic activity in the cell cultures, but 5moU‐mRNA did so to a significantly lesser extent than all other mRNAs. Senescence was not induced by unmodified or 5moU‐mRNA, suggesting that the observed reduction in metabolic activity was rather related to reduced cell proliferation and protein biosynthesis (Figure 2c).

Assuming that protein expression is linked to cell metabolic activity, it seems surprising that the peak protein expression of m1Ψ‐mRNA matches or sometimes even exceeds that of 5moU‐mRNA. This implies that metabolic activity impairment may not the only factor that determines how much protein is produced from IVT‐mRNA: Although 5moU‐mRNA triggered the least immune response (Figure 2d) and impaired the cells' metabolic activity the least (Figure 2b), m1Ψ is known to additionally increase translation efficacy at the ribosome, thus resulting in high peak production of protein.^[^ ^23^ ^]^ Thus, m1Ψ‐ and 5moU‐mRNA both yield high levels of protein production, but they do so via different mechanisms: 5moU thanks to minimal immunogenicity and m1Ψ through a combination efficient peak translation and low‐moderate immunogenicity.

Aside from protein biosynthesis, the proposed mechanism underlying the cellular responses toward IVT‐mRNA is an anti‐viral type‐I interferon response, as confirmed by the induction of the type‐I interferon IFN‐β, especially by U‐mRNA but also to a lesser degree by Ψ and m1Ψ (Figure 2d). Furthermore, we observed that the cytokine IL‐6, which is naturally produced by BMSCs, was upregulated by U‐mRNA and Ψ‐mRNA (Figure 2e). This led us to the question whether the entire secretion pattern might be affected by mRNA transfection.

To investigate this, we analyzed cell culture supernatants of untransfected and mRNA‐transfected cells (or conditioned media) using a protein array of >250 proteins (Figure 3). Importantly, we included all 3 modified mRNAs into this experiment to understand whether potential phenotypic changes would be qualitatively different for the different mRNA chemistries or only vary their intensity. Also, we tested supernatants of cells from two different donors, to estimate to what extent any observed effects can be generalized across individuals.

At first glance, when looking at the raw detection values of the most abundant factors in the cell culture supernatants, a small cluster of cytokines including CXCL10, CXCL11 and CCL20 appeared conspicuously upregulated in both U‐ and Ψ‐mRNA transfected cells (Figure 3a). Analyzing the fold change of each analyte across experimental groups painted a more nuanced picture, where differences between the U and Ψ groups, but also some variance between donors became evident (Figure 3b). Additionally, a cluster of analytes appeared upregulated primarily by U‐mRNA, but less so by Ψ‐mRNA, suggesting the possibility of a non‐linear component in the regulation of immune responses by unmodified versus modified mRNA. Regarding the difference between absolute and relative change in analyte levels (Figure 3a vs. 3b), it should also be noted, that for analytes which are expressed at very low levels, even minimal changes in absolute expression could lead to a many‐fold relative change, thus somewhat blurring the picture of which of the observed relative changes are occurring at relevant absolute levels. For this reason, both the absolute expression (Figure 3a) and the fold change in expression (Figure 3b) are shown. For example, while CXCL10, CXCL11 and CCL20 were expressed at high absolute levels in the U‐ and Ψ‐mRNA groups, CXCL9 also showed a fold change but was lowly expressed in all conditions. IL‐8 on the other hand was very highly expressed in all conditions despite showing a relative change. Caspases were also found to be upregulated in this array, but are found predominantly in the cytoplasm, so their raw values in the supernatant were expectedly low, making their activity within cells (Figure 2a) the more relevant readout for apoptosis.

To understand to what extent the different observed differences contributed to the overall variance in the data, a principal component analysis was conducted (Figure 3c). First, it revealed that the effect of mRNA transfections outweighed the difference between biological donors, since the spatial relationship between group scores was similar for both donors and formed two parallel lines orthogonal to the direction of PC2. Second, it showed that the paracrine shifts induced by the different mRNA chemistries differed quantitatively rather than qualitatively, as the group scores for each donor arranged into a line along the most dominant vector (CXCL10). It is important to note that scores for the 5moU‐mRNA group and the untransfected group overlapped in both donors, indicating that 5moU‐mRNA did not affect the paracrine phenotype of BMSCs at all. This is in line with the lack of IFN‐β induction by this mRNA and suggests that 5moU‐mRNA allows to transiently overexpress a protein of choice practically without leaving a recognizable footprint on the modified cell. In our view this finding highlights the unique potential of IVT mRNA technology to be used for a broad range of therapeutic indications with minimal side effect.

Considering the potentially endless variety of target proteins that may be encoded by mRNA drugs, it was important to understand whether the characterized cellular responses toward EGFP‐encoding mRNA are specific for this mRNA sequence or could be generalized for all mRNAs, regardless of which protein they encode. To answer this, we tested mCherry‐encoding and IL‐10‐encoding mRNA alongside the EGFP‐encoding mRNA (Figure 6). For all three mRNAs the relationships between mRNA chemistry and protein expression and between mRNA chemistry and immune activation matched closely. Even raw values of induced IFN‐β and CXCL10 secretion were in the same range, demonstrating that an mRNA's function as an immune receptor ligand is largely independent from the protein it encodes. One sequence‐related parameter which may of course impact impact immune recognition is of course the uridine content of the sequence. Thess et al. have shown that by reducing uridine content of IVT‐mRNA through codon optimization, immune activation can also be reduced, although of course uridine cannot be completely eliminated from mRNA.^[^ ^43^ ^]^ For natural, uridine‐containing coding sequences like the ones we have tested, this mechanism is of secondary importance and does not contradict the importance of uridine chemical modifications or their relative potency in reducing immune activation.

Regarding the relationship between mRNA chemistry and protein expression, it may appear that the production of IL‐10 (Figure 6c) is shorter than for EGFP or mCherry (Figure 6a and b). However, it is important to note that while IL‐10 is being constantly secreted, the two fluorescent proteins accumulate in the cytoplasm and thus are detectable even after production has ceased. This is an important reminder that not only the duration of protein production, but also the half‐life and fate of the produced protein of interest can determine the duration of a therapeutic effect exerted by an mRNA drug. In the case of a transmembrane protein, its presence would additionally depend on the turnover of the cell membrane.

Because immune recognition of mRNA depends on recognition of the chemical identity of the macromolecule rather than the specific base sequence, as detailed above, we used EGFP mRNA to characterize cellular responses. As EGFP is bioinert and remains inside the cell, it allows to study the secretion pattern of cells without interference and does not influence the cell phenotype by autocrine feedback. However, we did validate the immune response and CXCL10 induction by differently nucleotide‐modified mRNA using IL‐10 mRNA, which confirmed our findings findings with EGFP mRNA (Figure 5). Additionally, we included time as a variable, by measuring a time course of IFN‐β and CXCL10 secretion after transfection (Figure 5c,d). IFN‐β secretion peaked early and faded out almost completely on day 2. CXCL10 on the other hand peaked on day 2 (for Ψ‐ and m1Ψ‐mRNA) was detectable until day 4, which fits the proposed mechanism that the observed paracrine shifts are secondary effects of a type‐I interferon‐driven response. Secretion of mRNA‐induced CXCL10 was several orders of magnitude lower but lasted almost as long as the secretion of mRNA‐encoded IL‐10. CXCL10 secretion from U‐ versus Ψ‐transfected cells was similar in all experiments (cf. Figure 3b, Figure 5c) and showed no significant difference for any tested IVT‐mRNA sequence in Figure 6d–f. This may seem counterintuitive given the consistently higher interferon response activation by U‐mRNA. However, this along with the faster decline of CXCL10 secretion in U‐mRNA transfected cells compared to the Ψ and m1Ψ groups (Figure 5d) might be explained by apoptosis of cells transfected with unmodified mRNA, as opposed to those transfected with any of the modified variants (Figure 2a). It could thus be said that although antiviral mechanisms are triggered stronger in U‐transfected cells than in Ψ‐transfected cells, this does not necessarily translate to higher chemokine production in a linear manner, because the survival of U‐transfected cells is reduced. 5moU‐modified IL‐10 mRNA did not induce any IFN‐β or CXCL10 secretion.

Although the presence of cytokines and growth factors, as detected by ELISA or protein arrays, can be suggestive of a functional biological process, it is important to acknowledge that (I) these assays do not cover every protein produced by the cell and (II) the derived assumptions about function do not account for synergistic or antagonistic crosstalk in responder cells. Since the secretome pattern shift induced by IVT mRNA implied a pro‐inflammatory and immune cell recruiting function for mRNA‐transfected cells, we decided to confirm this with functional immune assays (Figure 4).

Using a lymphocyte migration assay previously established in our group to study the CXCR3 system,^[^ ^35^ ^]^ we demonstrated that primary human T cells (CD4^+^ and CD8^+^) were indeed attracted by supernatants from U‐mRNA transfected BMSCs but not from untransfected or 5moU‐transfected cells (Figure 4b). Recombinant CXCL11 at a concentration of 100 ng mL^−1^ was used as a positive control because it was previously found to induce the strongest CXCR3‐mediated migration among the three ligands.^[^ ^35^ ^]^ The fact that CD19^+^ B cells were strongly attracted by the secretome of U‐mRNA transfected cells but not by recombinant CXCL11 suggests that other factors in the secretome were responsible for B cell migration. In other studies, B cell migration was shown to be mediated by the chemokines CCL19, CCL20, CCL21, CXCL12 and CXCL13.^[^ ^44^, ^45^ ^]^ Out of those, CCL20 was also included in our protein array and was among the most strongly upregulated factors by immunogenic mRNA, both regarding fold change and increase in raw value (Figure 3b and a respectively).

Next, we asked how the lymphocytes would interact once attracted by the transfected BMSCs. BMSCs are known to reduce the expansion of T lymphocytes, and this is also often attributed to their mode of action in therapeutic applications of this cell type.^[^ ^24^, ^36^ ^]^ U‐mRNA transfected BMSCs still reduced T cell proliferation but did so significantly less effectively than untransfected cells (Figure 4c). Transfection with any of the modified mRNAs did not affect T cell proliferation compared to untransfected BMSCs.

Considering local application of mRNA drugs in patients these findings point toward an opportunity to deliberately exploit the immune stimulatory and lymphocyte recruiting effects of IVT mRNA in redirecting the immune system. As mentioned above, the classification of mRNA immune activation as favorable or unfavorable depends on the intended application.^[^ ^3^, ^40^ ^]^ Importantly however, we show that the intensity of this effect can be gradually tailored by replacing uridine in the IVT reaction with the appropriate derivate: Ψ for moderate immune activation and m1Ψ or 5moU for low or very low immune activation. We have previously shown that activation of the CXCR3 chemokine system plays a role in the immune response toward solid tumors and is predictive of therapy response.^[^ ^35^ ^]^ Thus, the ability to engineer an immune activation of bespoke intensity paves a way toward exploiting mRNA‐induced CXCR3 ligand secretion in the context of tumor therapy, possibly to boost other regimens, such as CXCR3‐expressing CAR‐T cells against solid tumors.

As an example of a therapeutically relevant secreted target, we overexpressed IL‐10. mRNA overexpression of IL‐10 has been reported before in BMSCs and improved the cells' immunomodulatory potency in preclinical BMSC transplantation. In the first report by Levy et al.,^[^ ^6^ ^]^ which was published before m1Ψ^[^ ^22^ ^]^ and 5moU^[^ ^13^ ^]^ were introduced, Ψ‐mRNA was used. The study by Zhang et al.^[^ ^25^ ^]^ was conducted with unmodified mRNA. In both cases the immunomodulatory potential of BMSCs was improved by the IL‐10 overexpression. However, the impact of mRNA transfection on the cells' natural fitness and immunosuppressive signaling was not considered in either study. Thus, we hypothesized that the innate immune response triggered by immunogenic IL‐10 mRNA might at least partially antagonize the intended anti‐inflammatory effect. Indeed, supernatants from cells transfected with unmodified IL‐10 mRNA were less effective at suppressing TNF‐α than any of the modified mRNA groups (Figure 5e). Overall, however, all IL‐10 overexpressing cells were more effective than untransfected cells. This suggests that while the immunosuppressive effect of IL‐10 outweighs the induction of a proinflammatory secretome (Figure 3), using an IVT mRNA with minimal immune activation for anti‐inflammatory applications bolsters the intended effect and might for instance help reduce the required mRNA dose.

Despite having used freshly drawn human donor blood and selecting well‐established immuno‐assays which mirror our clinical study immunophenotyping protocols,^[^ ^35^, ^38^ ^]^ questions about the behavior of mRNA‐transfected cells and their interactions with immune cell populations in vivo remain open and should be investigated using appropriate animal models. Addressing this question is outside the scope of this study, as it would require mirroring the cells, mRNAs, protein arrays and underlying molecular pathways from this study in a rodent system and ‐most importantly‐ testing them in an appropriate immune competent animal model. However, substantial differences between the human and rodent immune systems impede the transferability of study results between both organisms,^[^ ^46^ ^]^ leading us to focus on investigations in a human platform.

## 4 Conclusions

We systematically compared the innate immune activation by unmodified and Ψ‐, m1Ψ‐, and 5moU‐modified IVT mRNAs and investigated their impact on the phenotype and function of transfected cells. Unmodified mRNA triggered an anti‐viral‐like IFN‐β response in transfected cells and induced a pro‐inflammatory paracrine pattern marked by the secretion of lymphocyte‐attracting chemokines. This mRNA‐induced secretome pattern recruited both T and B lymphocytes, which bears implications and opportunities for intentionally exploiting this effect in mRNA drug design.

Importantly, replacing uridine with 5‐methoxyuridine (5moU) was sufficient to completely reduce the immune activation and related functional effects.

Our findings demonstrated that IVT‐mRNA has not one, but two functions which can be uncoupled, controlled, and quantitatively adjusted: the protein coding function and the less obvious immune‐stimulatory shift of transfected cells and their immune environment.

We showed that the extent of this immune stimulatory shift varies quantitatively between unmodified, Ψ‐, m1Ψ‐, and 5moU‐modified IVT mRNA (from highest immune activation to lowest). Thus, the intensity of mRNA's immune stimulatory function can be gradually and predictably tailored to the desired inflammatory (cancer or viral vaccination) or non/anti‐inflammatory (tolerance, protein replacement, regeneration) application.

To illustrate this, we showed that both the anti‐proliferative effect of BMSCs on T cells, and the immunosuppressive effect of IL‐10 mRNA could be reinforced through the appropriate uridine modification.

## 5 Experimental Section

### Cell Culture

Human bone marrow mesenchymal stromal/stem cells (BMSCs) from patients undergoing hip surgery at Charité—Universitätsmedizin Berlin were supplied by the BIH Center for Regenerative Therapies (BCRT) core facility “Cell Harvesting” as previously stated.^[^ ^47^ ^]^ Written informed consent was given, and ethics approval was obtained from the institutional review board (IRB)/ local ethics committee of the Charité— Universitätsmedizin Berlin (number of approval: EA2/089/20). As described previously, DMEM low glucose with 10% v/v fetal bovine serum (FBS), and 1% v/v Glutamax (Thermo Fisher Scientific Inc., Waltham, MA) and 1% v/v penicillin/streptomycin (both Biochrom AG) was used to culture BMSCs.^[^ ^16^ ^]^ The cells were cultured at 37 °C in a humidified 5% CO_2_ atmosphere. Media were changed at least 3x/ week and BMCSs passed before reaching confluence. Experiments were performed using BMSCs from at least two different donors, unless stated otherwise.

Blood was drawn from consenting adult donors (Charité ethics committee approval EA4/091/19). The blood was immediately diluted 1:1 (v:v) with PBS, added slowly on top of 15 mL Pancoll (density 1.077 g mL^−1^; Pan Biotech, Aidenbach, Germany) without mixing the two phases and centrifuged at room temperature, 800 * g for 20 min using the slowest possible break setting. After centrifugation the layer containing the PBMCs was carefully transferred into PBS, washed, and resuspended in RPMI1640 medium (Thermo Fisher Scientific).

### In Vitro Transcription of EGFP and IL‐10 mRNA

The plasmid vector template pRNA2‐(A)128^[^ ^48^ ^]^ (gift from Stephen Ikeda; Addgene plasmid # 174 006), was used as a template for IVT. The original plasmid contains a 5′‐UTR, the sequence for EGFP (Figure S1, Supporting Information), a human β‐globin 3′‐UTR, flanked by a T7 promoter (upstream) and a 128‐base polyadenine tail on the 3′ end. An insert containing the IL‐10 (or mCherry) coding sequence (Figure S1, Supporting Information) was designed in silico, ordered from Integrated DNA Technologies (Coralville, IA) and cloned into the pRNA2‐(A)128 vector using restriction endonucleases HindIII and NotI (New England Biolabs, Ipswich, MA) to replace the EGFP coding sequence. The fragments of interest from all three plasmids spanning the T7 promoter and poly(A) tail were amplified using PCR and purified (PCR Clean‐up kit; Macherey‐Nagel, Düren, Germany) to serve as a linear DNA template for IVT. The primers spanning the T7 promoter, and the 128‐base poly(A) tail are shown in Figure S2 (Supporting Information).

The TranscriptAid T7 Kit (Thermo Fisher Scientific) was used for in vitro transcription, as previously described.^[^ ^16^ ^]^ Briefly, nucleotide triphosphates (100 mM) and anti‐reverse cap analog (ARCA; 30 mM) were added to the IVT reaction. For synthesis of nucleotide‐modified mRNA, unmodified uridine triphosphate was fully replaced with chemically modified nucleotides (Ψ, m1Ψ, 5moU; 100 mM; all Jena Bioscience, Germany). mRNA integrity was verified by running 2 uL of mRNA with 5 uL loading dye (included in the IVT kit) on a 0.9% RNAse‐free agarose gel. mRNA concentration was adjusted to 1 mg mL^−1^ using UV–vis‐spectroscopy (NanoDrop 1000; Peqlab, Erlangen, Germany). IVT yields were similar regardless of what uridine derivate was used (**Table** **1**).

**Table 1:** IVT yields for two exemplary batches of mRNA.

Target protein	Modification	Reaction scale [n‐fold]	Concentration [ng/µL]	End volume [µL]	Total IVT yield [µg]
IL‐10	U	1	1034.2	48	49.64
IL‐10	PU	1	1085.7	45	48.86
IL‐10	M1PU	1	1095.9	50	54.8
IL‐10	5moU	1	1027.9	52	53.45
GFP	U	2	1051.3	100	105.13
GFP	PU	2	1064	95	101.08
GFP	M1PU	2	1065.7	100	106.57
GFP	5moU	2	1093.3	95	104.96

### mRNA Transfection

To form mRNA‐lipoplexes, IVT‐mRNA was mixed with Lipofectamine MessengerMAX (LMM; Thermo Fisher Scientific) at a 1:2 ratio (w:v) in Opti‐MEM medium (Thermo Fisher Scientific). Lipoplexes were gently added into BMSC culture wells. Experiments were conducted using 125 ng cm^−2^ mRNA per culture area (2.5 * 10^4^ cells). To control for effects of the transfection reagent, a control group treated only with the carrier lipid LMM and Opti‐MEM were included in all experiments in addition to the untransfected control.

### Fluorescence Microscopy

An inverted fluorescent microscope (ELIPSE Ti–U equipped with pE‐300lite LED light source; Nikon, Düsseldorf, Germany) along with NIS‐Elements imaging software version 4.51 (Nikon) were used to image EGFP and mCherry fluorescence in BMSCs.

### Proliferation Assay

Cells were seeded at a density of 5 * 10^3^ cells cm^−2^ culture area on day −1 and allowed to proliferate. On days 1, 3 and 5 post transfection, the cells were incubated for 45 min at 37 °C in a 1:10 v/v mix of PrestoBlue reagent (Thermo Fisher Scientific) and culture medium. To quantify the cells' mitochondrial metabolic activity, fluorescence was measured at 570 nm using a plate reader (Tecan, Männedorf, Switzerland).

### Caspase Activity

To measure the activity of caspases 3 and 7 in BMSCs, 24 h post‐transfection the cells were incubated for 1 h in a 1:2 v/v mix of Caspase‐Glo 3/7 3D (Promega, Madison, WI) reagent and culture medium, before quantifying luminescence (GloMax; Promega), according to the user manuals.

### β‐Galactosidase Staining

Staining of senescent cells was performed using the Senescence β‐Galactosidase Staining Kit (Cell Signaling Technology, Danvers, MA). Briefly, 24 well plates with transfected or untransfected BMSCs were fixed on day 5 (or 7 post) transfection (Figure S3, Supporting Information). After washing the plate gently with PBS, staining solution containing 1 mg mL^−1^ X‐gal and equilibrated to pH 6.0 was added. The plate was incubated for 24 h in a dry incubator incubator at 37 °C and imaged using an inverted microscope (ELIPSE Ti–U, Nikon). Senescence 5 days post‐transfection was quantified in Image J by counting β‐galactosidase‐positive (blue) cells per field.

### Proteomics

IL‐6, IFN‐β, CXCL10 and IL‐10 were measured in cell culture supernatants using ELISA (R&D Systems, Minneapolis, MN) according to the manufacturer's instructions. Unconditioned DMEM +10% FCS from the same batch as used in the respective experiment was used as a blank. To assess paracrine pattern shifts of mRNA‐treated cells, the concentrations of 266 proteins in BMSC‐conditioned media were measured using an Olink Target 96 proximity extension assay (Olink Bioscience, Uppsala, Sweden), using the procedure, detection limits and analysis method previously described.^[^ ^16^, ^47^ ^]^ Detailed information on this assay, can be found on the company's website (www.olink.com/downloads). The assay was carried out in biological duplicates, testing two different cell donors. Heatmaps were generated using the ComplexHeatmap package, while biplots were generated with the prcomp function and factoextra package version 1.0.7. All analyses were performed using R version 4.1.1, available free online at https://www.r‐project.org.

### Immune Cell Migration

Freshly isolated human PBMC (1 * 10^6^) from peripheral blood of healthy donors were seeded into the upper chamber of a transwell plate (HTS Transwell‐24‐well, 3.0 µm Pore, Corning). BMSC‐conditioned medium (CM) from EGFP‐mRNA (U or 5moU) transfected or untransfected BMSC was added to the lower chamber. Recombinant human CXCL11 (1 ng mL^−1^, 100 ng mL^−1^, BioLegend) served as a positive control, while unconditioned BMSC media served as a blank. The migration assay was conducted in a humidified incubator at 37 °C, 5% CO_2_ for 3 h. The absolute number of migrated CD4^+^ and CD8^+^ T cells and CD19^+^ B cells were determined using a CytoFLEX LX flow cytometer (Beckman Coulter). A chemotactic index (CI) was calculated, as described before^[^ ^35^ ^]^ based on the absolute number of migrated cells with conditioned medium from transfected BMSC, normalized to the absolute number of migrated cells without conditioned media (blank). The assay was conducted with blood from n = 5 biological donors, and repeated using different batches of BMSC‐CM.

### Immune Cell Proliferation

BMSCs were seeded in a flat bottom 96‐well plate (8 * 10^3^ cells per well) and transfected with mRNA 24 h later. PBMCs were stained with 5 µM carboxy‐fluorescein‐succinimidyl ester (CFSE; BioLegend), as described before.^[^ ^36^ ^]^ 2 h after transfection, the plate containing BMSCs was washed gently with warm medium to remove any excess mRNA and 2 * 10^5^ CFSE‐stained PBMCs in RPMI1640 (Thermo Fisher Scientific) supplemented with 10% FCS were added. To induce proliferation, T cells were stimulated using anti‐CD3 (0.125 µg mL^−1^) and anti‐CD28 (0.25 µg mL^−1^) antibodies (BD Biosciences). Unstimulated wells served as a control for low proliferation, while wells with stimulated PBMCs but without BMSCs were included as a control for high proliferation. The co‐culture plates were kept in a humidified incubator at 37 °C, 5% CO_2_ for 5 days, after which T cells were stained and quantified using flow cytometry (see below). CFSE was diluted with every cell division, resulting in a measurable decrease in CFSE‐intensity. The experiment was performed in triplicates.

### Flow Cytometry

EGFP‐mRNA or mCherry‐mRNA expression in BMSCs was quantified by flow cytometry at the time points indicated in the figures. Detached and washed cells were stained with DAPI and measured using a MACSQuant VYB flow cytometer (Miltenyi Biotec). Cell populations were identified as follows: The BMSC population was selected in a forward (FSC) versus side scatter (SSC) plot and FSC‐area was plotted against FSC‐height (FSC‐A against FSC‐H) to identify single cells. Cell viability was measured by quantifying the DAPI+ populations of each sample and EGFP+ (or mCherry+) cells were quantified within the live populations in relation to untransfected control samples.

PBMCs were stained using fluorophore‐conjugated human anti‐CD3 (OKT3; SP34‐2), ‐CD4 (SK3; OKT4), ‐CD8 (RPA‐T8; QA18A37), ‐CD19 (SJ25C1) (all from BioLegend) and the LIVE/DEAD Fixable Near‐IR Dead Cell Stain Kit (L/D; Invitrogen). Cell populations were identified according to the gating strategy shown in Figure S4 (Supporting Information).

### Sepsis Attenuation Assay

Blood was drawn from n = 4 healthy donors and diluted 1:10 in RPMI1640 (Thermo Fisher Scientific) or BMSC‐CM diluted in RPMI. Endotoxin (lipopolysaccharide; LPS) from E. coli serotype O55:B5 was added at a concentration of 1 ng mL^−1^ to induce a septic response. After 4 h incubation (37 °C, 5% CO_2_), the diluted blood was centrifuged for 5 min at 300 * g and the fluid phase was collected for ELISA measurements.

### Statistical Analysis

Normalization of raw values was carried out in some cases to facilitate comparisons: In Figure 2a,b, values were normalized to the mean value of the untransfected group (on day 1). In Figure 3b values for each analyte were normalized to the mean value across all experimental groups. In Figure 4a the number of migrated cells in experimental groups was, normalized to the absolute number of migrated cells in blank (unconditioned) medium to calculate the chemotactic index. Values are depicted as mean ± standard derivative (SD), unless stated otherwise in the respective figure legend. Experiments were conducted using cells from at least two biological donors, unless indicated otherwise. Sample sizes for each experiment are included in the respective figure legends. One way ANOVA (comparison at a single timepoint), two‐way ANOVA (time courses) or mixed‐effects analysis (if values were missing) with Tukey's post‐hoc test for multiple comparisons or Dunnett's post‐hoc test for many‐to‐one comparisons was used to test for significant differences between groups, as detailed in the figure legends. Levels of statistical significance were set at ∗p < 0.05, ∗∗p < 0.01, ∗∗∗p < 0.001. Statistical analysis was carried out in GraphPad Prism 9.0 (GraphPad Software Inc., USA).

## Conflict of Interest

The authors declare no conflict of interest.

## Supporting information

Supporting Information

## Data Availability

The data that support the findings of this study are available from the corresponding author upon reasonable request.
