# Supplementary material for: In Vitro Transcribed mRNA Immunogenicity Induces Chemokine‐Mediated Lymphocyte Recruitment and Can Be Gradually Tailored by Uridine Modification
Source: Adv Sci (Weinh). 2024 Mar 16;11(21):2308447. doi: 10.1002/advs.202308447 (PMC11151007; doi:10.1002/advs.202308447)
Supplement: Supplementary file 1 — Supporting Information [file ADVS-11-2308447-s001.pdf]

ADVANCED SCIENCE
Open Access

Supporting Information

for *Adv. Sci.*, DOI 10.1002/advs.202308447

In Vitro Transcribed mRNA Immunogenicity Induces Chemokine-Mediated Lymphocyte Recruitment and Can Be Gradually Tailored by Uridine Modification

Norman M. Drzeniek, Nourhan Kahwaji, Samira Picht, Ioanna Maria Dimitriou, Stephan Schlickeiser, Hanieh Moradian, Sven Geissler, Michael Schmueck-Henneresse, Manfred Gossen* and Hans-Dieter Volk*

SUPPLEMENTARY INFORMATION

In Vitro Transcribed mRNA Immunogenicity Induces Chemokine-Mediated Lymphocyte Recruitment and Can Be Gradually Tailored by Uridine Modification

Norman M. Drzeniek*, Nourhan Kahwaji, Samira Picht, Ioanna Maria Dimitriou, Stephan Schlickeiser, Hanieh Moradian, Sven Geißler, Michael Schmueck-Henneresse, Manfred Gossen*, Hans-Dieter Volk

*co-corresponding authors: norman.drzeniek@charite.de, manfred.gossen@hereon.de

Affiliations:
Charité – Universitätsmedizin Berlin, corporate member of Freie Universitaet Berlin and Humboldt-Universitaet zu Berlin, Institute of Medical Immunology, Augustenburger Platz 1, 13353 Berlin, Germany: NM Drzeniek, S Schlickeiser, H-D Volk

Berlin Institute of Health at Charité – Universitätsmedizin Berlin, BIH Center for Regenerative Therapies (BCRT), Föhrer Straße 15, 13353 Berlin, Germany: NM Drzeniek, N Kahwaji, S Picht, IM Dimitriou, S Schlickeiser, S Geißler, M Schmueck-Henneresse, H-D Volk

Berlin-Brandenburg School for Regenerative Therapies (BSRT; graduate school 203 of the German Excellence Initiative), Augustenburger Platz 1, 13353 Berlin: S Picht, IM Dimitriou

Berlin Institute of Health at Charité – Universitätsmedizin Berlin, Julius Wolff Institute (JWI), Augustenburger Platz 1, 13353 Berlin, Germany: IM Dimitriou, S Geißler

Charité – Universitätsmedizin Berlin, corporate member of Freie Universität Berlin and Humboldt-Universität zu Berlin, Berlin Center for Advanced Therapies (BeCAT), Augustenburger Platz 1, 13353 Berlin, Germany: S Geißler, M Schmueck-Henneresse, H-D Volk

CheckImmune GmbH, Campus Virchow Klinikum, Augustenburger Platz 1, 13353, Berlin, Germany: S Schlickeiser, H-D Volk

Freie Universität Berlin, Department of Biology, Chemistry, Pharmacy, Institute of Chemistry and Biochemistry, Thielallee 63, 14195 Berlin, Germany: IM Dimitriou

Institute of Active Polymers, Helmholtz-Zentrum Hereon, Kantstraße 55, 14513 Teltow, Germany: H Moradian, M Gossen

Berlin-Brandenburg Center for Regenerative Therapies (BCRT), Augustenburger Platz 1, 13353 Berlin, Germany: H Moradian, M Gossen

Orcid ID numbers:
Norman M Drzeniek https://orcid.org/0000-0001-6562-2351
Nourhan Kahwaji https://orcid.org/0000-0002-9326-8529
Samira Picht https://orcid.org/0000-0002-7761-5711
Ioanna Maria Dimitriou https://orcid.org/0000-0002-2299-2042
Stephan Schlickeiser https://orcid.org/0000-0003-3142-2890
Hanieh Moradian https://orcid.org/0000-0002-8729-9425
Sven Geissler https://orcid.org/0000-0002-8750-6324
Michael Schmueck-Henneresse https://orcid.org/0000-0001-5964-9179
Manfred Gossen https://orcid.org/0000-0002-1761-4063
Hans-Dieter Volk https://orcid.org/0000-0002-7743-6668

List of Supplementary Information:

Figure S1. Open reading frames for EGFP and IL-10.

Figure S2. PCR primer sequences.

Figure S3. β-galactosidase staining of mRNA-transfected cells on days 5 and 7

Figure S4. Representative gating strategy for flow cytometric analysis of migrated cell populations.

Enhanced GFP	ATGGTGAGCAAGGGCGAGGAGCTGTTCACCGGGGTGGTGCCCATCCTGGTCGAGCTG<br>GACGGCGACGTAAACGGCCACAAGTTCAGCGTGTCCGGCGAGGGCGAGGGCGATGC<br>CACCTACGGCAAGCTGACCCTGAAGTTCATCTGCACCACCGGCAAGCTGCCCGTGCC<br>CTGGCCCACCCTCGTGACCACCCTGACCTACGGCGTGCAGTGCTTCAGCCGCTACCCC<br>GACCACATGAAGCAGCACGACTTCTTCAAGTCCGCCATGCCCGAAGGCTACGTCCAG<br>GAGCGCACCATCTTCTTCAAGGACGACGGCAACTACAAGACCCGCGCCGAGGTGAAG<br>TTCGAGGGCGACACCCTGGTGAACCGCATCGAGCTGAAGGGCATCGACTTCAAGGAG<br>GACGGCAACATCCTGGGGCACAAGCTGGAGTACAACTACAACAGCCACAACGTCTAT<br>ATCATGGCCGACAAGCAGAAGAACGGCATCAAGGTGAACTTCAAGATCCGCCACAA<br>CATCGAGGACGGCAGCGTGCAGCTCGCCGACCACTACCAGCAGAACACCCCCATCGG<br>CGACGGCCCCGTGCTGCTGCCCGACAACCACTACCTGAGCACCCAGTCCGCCCTGAG<br>CAAAGACCCCAACGAGAAGCGCGATCACATGGTCCTGCTGGAGTTCGTGACCGCCGC<br>CGGGATCACTCTCGGCATGGACGAGCTGTACAAGTAA
human interleukin-10	ATGCACAGCTCAGCACTGCTCTGTTGCCTGGTCCTCCTGACTGGGGTGAGGGCCAGCC<br>CAGGCCAGGGCACCCAGTCTGAGAACAGCTGCACCCACTTCCCAGGCAACCTGCCTA<br>ACATGCTTCGAGATCTCCGAGATGCCTTCAGCAGAGTGAAGACTTTCTTTCAAATGAA<br>GGATCAGCTGGACAACTTGTTGTTAAAGGAGTCCTTGCTGGAGGACTTTAAGGGTTA<br>CCTGGGTTGCCAAGCCTTGTCTGAGATGATCCAGTTTTACCTGGAGGAGGTGATGCCC<br>CAAGCTGAGAACCAAGACCCAGACATCAAGGCGCATGTGAACTCCCTGGGGGAGAA<br>CCTGAAGACCCTCAGGCTGAGGCTACGGCGCTGTCATCGATTTCTTCCCTGTGAAAAC<br>AAGAGCAAGGCCGTGGAGCAGGTGAAGAATGCCTTTAATAAGCTCCAAGAGAAAGG<br>CATCTACAAAGCCATGAGTGAGTTTGACATCTTCATCAACTACATAGAAGCCTACAT<br>GACAATGAAGATACGAAACTGA
mCherry	ATGGTCAGCAAGGGAGAGGAAGATAATATGGCGATCATCAAAGAGTTTATGAGATTT<br>AAGGTGCACATGGAAGGAAGCGTTAATGGTCATGAGTTTGAAATCGAAGGCGAAGG<br>CGAAGGAAGACCGTATGAAGGCACACAGACGGCTAAACTTAAGGTCACAAAAGGCG<br>GACCGCTTCCATTCGCGTGGGATATTCTTTCACCGCAATTTATGTATGGTTCTAAAGC<br>CTATGTGAAACATCCTGCGGATATTCCTGACTACCTTAAACTGTCTTTCCCGGAAGGA<br>TTTAAATGGGAACGCGTCATGAACTTCGAAGATGGCGGCGTTGTTACGGTGACGCAG<br>GATTCATCACTGCAAGATGGAGAATTTATTTATAAAGTTAAACTGCGCGGCACAAAC<br>TTTCCGTCAGACGGACCTGTCATGCAGAAGAAAACGATGGGCTGGGAAGCCAGCAGC<br>GAGAGAATGTACCCGGAGGACGGAGCACTTAAAGGCGAAATCAAGCAACGCCTGAA<br>GCTGAAAGATGGAGGCCATTATGATGCCGAGGTCAAGACGACATACAAAGCTAAGA<br>AACCGGTACAATTACCTGGAGCATACAACGTCAATATCAAGCTGGATATTACGTCAC<br>ATAATGAAGACTATACGATTGTAGAGCAATATGAAAGAGCAGAGGGAAGACACTCT<br>ACAGGTGGAATGGACGAATTATACAAATAG

Figure S1. Open reading frames for EGFP, IL-10 and mCherry.

Forward (T7): GCTCTTAAGGCTAGAGTACTTAATACGACTCACTATAGGGAG

Reverse (Poly-A):
TTTTTTTTTTTTTTTTTTTTTTTTTTTTTTTTTTTTTTTTTTTTTTTTTTTTTTTTTTTTTTTTTTTTTT
TTTTTTTTTTTTTTTTTTTTTTTTTTTTTTTTTTTTTTTTTTTTTTTTTTTTTTTTTTGTTTAAACATT
TAAATGCAAT

Figure S2. PCR primer sequences.
These sequences were designed to amplify the linear DNA fragment needed as a template for IVT from the plasmid pRNA2-(A)128 (Addgene plasmid # 174006). They were used on the EGFP-, mCherry-, and IL-10 coding sequence-containing plasmid.

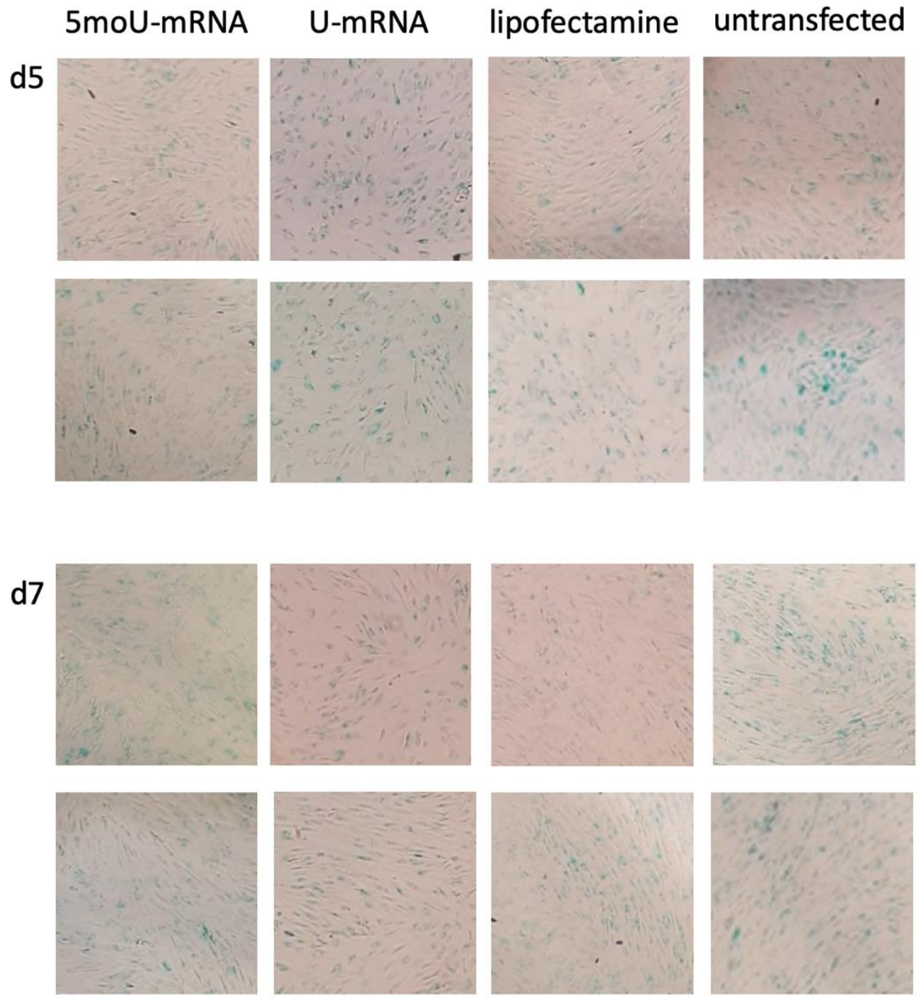

Figure S3. β-galactosidase staining of mRNA-transfected cells on days 5 and 7.

BMSCs were transfected with 5moU-modified or unmodified (U) mRNA. The experiment was performed in quadruplicates. Five and seven days after transfection, staining of senescent cells was performed using the Senescence β-Galactosidase Staining Kit (Cell Signaling Technology, Danvers, MA). No increase in β-galactosidase activity could be observed in the mRNA-transfected groups.

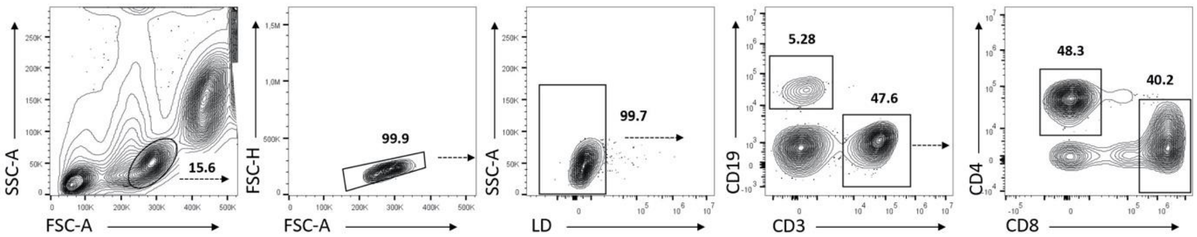

Figure S4. Representative gating strategy for flow cytometric analysis of migrated cell populations.

Migrated cells in the lower chamber of the migration trans-well were measured. The lymphocyte population was identified using the side and forward scatters. The population was gated for single cells and live cells. CD3+ T cells and CD19+ B cells were identified. Among T cells, CD4+ and CD8+ populations were identified.
